# Bleb plication: a minimally invasive repair method for a leaking ischemic bleb after trabeculectomy

**DOI:** 10.1038/s41598-020-72056-w

**Published:** 2020-09-11

**Authors:** Koichiro Sugimoto, Hiroshi Murata, Takehiro Yamashita, Ryo Asaoka

**Affiliations:** 1 Department of Ophthalmology, The University of Tokyo Graduate School of Medicine, 7-3-1 Hongo, Bunkyo-ku, Tokyo 1138655 Japan; 2 Department of Ophthalmology, Kagoshima University Graduate School of Medical and Dental Sciences, Kagoshima, Japan; 3 Department of Ophthalmology, Seirei Hamamatsu General Hospital, Hamamatsu, Shizuoka Japan; 4 Seirei Christopher University, Hamamatsu, Shizuoka Japan

**Keywords:** Outcomes research, Conjunctival diseases, Optic nerve diseases, Retinal diseases

## Abstract

Bleb leakage is a serious complication of glaucoma filtering surgery. This study describes the method and the results of a new repair method for ischemic bleb leaks. The subjects were consecutive eleven eyes of 11 patients with bleb leakage who underwent the bleb plication surgery. The bleb plication surgery consisted of two steps: 1) bleb needle redirection to float the conjunctiva away from the sclera as extensively as possible around the ischemic conjunctiva; and 2) multiple “O-shaped” sutures were applied between the non-ischemic conjunctiva just outside the ischemic conjunctiva and corneal limbus. The ischemic conjunctiva was not removed, but undermined beneath the advanced non-ischemic conjunctiva. This bleb plication method was repeated until the leakage was sealed. All patients were followed up for at least 6 months after final bleb plication. After final bleb plication, no recurrence of bleb leakage was observed. Moreover, ischemic changes were no longer observed in the advanced non-ischemic conjunctiva. Pre-operative and final intraocular pressure was 3.2 ± 4.1 and 11.9 ± 2.8 mmHg, respectively. This new repair method of bleb plication was safe and effective in sealing the leakage. The conjunctiva is not excised, and hence it does not run out.

## Introduction

Since proposed by Cairns et al. in 1968^1^, filtering surgery has been the most frequently performed surgery as the treatment of glaucoma worldwide. The outcome has been greatly improved with the adjunctive usage of Mitomycin C (MMC) and 5-fluorouracil (5-FU)^2–4^. However, various unfavorable ocular conditions, such as hypotony maculopathy, cataract, and bleb leakage, are experienced after surgery^5–11^. Among these, leakage from a bleb is a serious complication that is often observed in thin cystic and/or ischemic blebs, even after an extended period of time after surgery^12,13^. Treatment is essential without a delay, since it can lead to bleb infection and endophthalmitis. Treatment often starts with less invasive approaches, including pressure patching^14^, bandage contact lens^15^, collagen shield^16^, shell tamponade^17^, aqueous suppressants^18^, bleb needle redirection^19^, autologous blood injection^20^, and cyanoacrylate tissue glue^21^. Nonetheless, these treatments are not effective in a few cases, in whom surgical treatment is needed.

As reported by Burnstein et al.^18^, conjunctival advancement is a surgical treatment usually performed in such cases. However, this approach is often very invasive, in particular when there is a shortage of the conjunctiva, since the conjunctiva is advanced after the removal of the ischemic conjunctiva. Many other surgical techniques have been proposed, such as autologous conjunctival patch grafting^22^, amniotic membrane transplantation^23^, scleral patch graft^24^, corneal patch graft^25^, and fascia autograft^26^. Nonetheless, these treatments can be technically difficult and/or require special equipment.

Recently, we proposed a “bleb plication” technique to seal leakage from a bleb, as a case report^27^. This method was useful for a bleb leakage that could not be treated unless conjunctival advancement was performed. In addition, it is much less invasive than conjunctival advancement because the ischemic conjunctiva is not removed. Besides, Gupta et al. proposed incision-free minimally invasive conjunctival surgery (MICS) to seal a bleb leakage^28^. There are shared aspects between our bleb plication method and MICS; both attempt to seal bleb leakage by covering the ischemic bleb with the conjunctiva from the fornix side, without removing the ischemic conjunctiva. However, as described by the authors, MICS is not an option for eyes with severe conjunctival scarring or in which conjunctival mobilization is impractical, in contrast to the bleb plication method. In addition, the long-term outcome of MICS is not known, because most of the eyes were followed up only for short periods (< 2 months in 4 eyes and ≤ 3 months in 10 eyes among 14 eyes). In the present study, we report the outcome of the bleb plication method with a relatively long follow-up period (at least 6 months) in 11 consecutive eyes with bleb leakage, including those with severe conjunctival scarring and impractical conjunctival mobilization. The safety and efficacy of this method imply that it should be considered when treating an eye with leakage from a cystic bleb and/or ischemic bleb.

## Methods

This study was approved by the Research Ethics Committee of the Faculty of Medicine at the University of Tokyo. All patients provided Informed Consent for their information to be stored in the hospital database and used for research. This study was performed according to the tenets of the Declaration of Helsinki.

### Subjects

Eleven eyes of 11 consecutive patients with a bleb leakage were included in this study (Table 1). In all eyes, bleb leakage was not successfully treated using eye drops of aqueous suppressants and for dry eye, such as 2% rebamipide ophthalmic solution, and bleb needle redirection in some cases. Some of the patients in this study were also included in our previous case report^27^.

**Table 1:** Basic demographics of the patients.

Patient No	Age (years)	Sex	Eye	Diagnosis	Primary surgery	Antifibrotic agent	Onset of bleb leakage (months)	Previous treatment
1	64	M	L	POAG	Trabeculectomy	MMC	150	Eye drops + Needling
2	66	F	L	POAG	Trabeculectomy	MMC	162	Eye drops + Needling
3	86	M	L	PACG	Trabeculectomy	5-FU	288	Eye drops + Needling
4	67	M	L	NTG	Trabeculectomy	MMC	203	Eye drops + Needling
5	83	M	R	POAG	Trabeculectomy	MMC	141	Eye drops
6	43	F	L	NTG	Trabeculectomy	MMC	120	Eye drops + Needling
7	94	M	R	POAG	Trabeculectomy	5-FU	179	Conjunctival advancement Eye drops + Needling
8	75	M	R	POAG	Trabeculectomy	MMC	79	Eye drops + Needling
9	56	F	R	POAG	Trabeculectomy	MMC	174	Eye drops + Needling
10	57	F	L	NTG	Trabeculectomy	MMC	136	Eye drops
11	49	M	L	POAG	Trabeculectomy	MMC	149	Eye drops + Needling

POAG: primary open-angle glaucoma, PACG: primary angle-closure glaucoma, NTG: normal tension glaucoma, MMC: Mitomycin C, 5-FU: 5-fluorouracil, Eye drops: aqueous suppressants and eye drops for dry eye such as 2% rebamipide ophthalmic solution; Needling: bleb needle redirection.

### Bleb plication method

**Figure 1:**
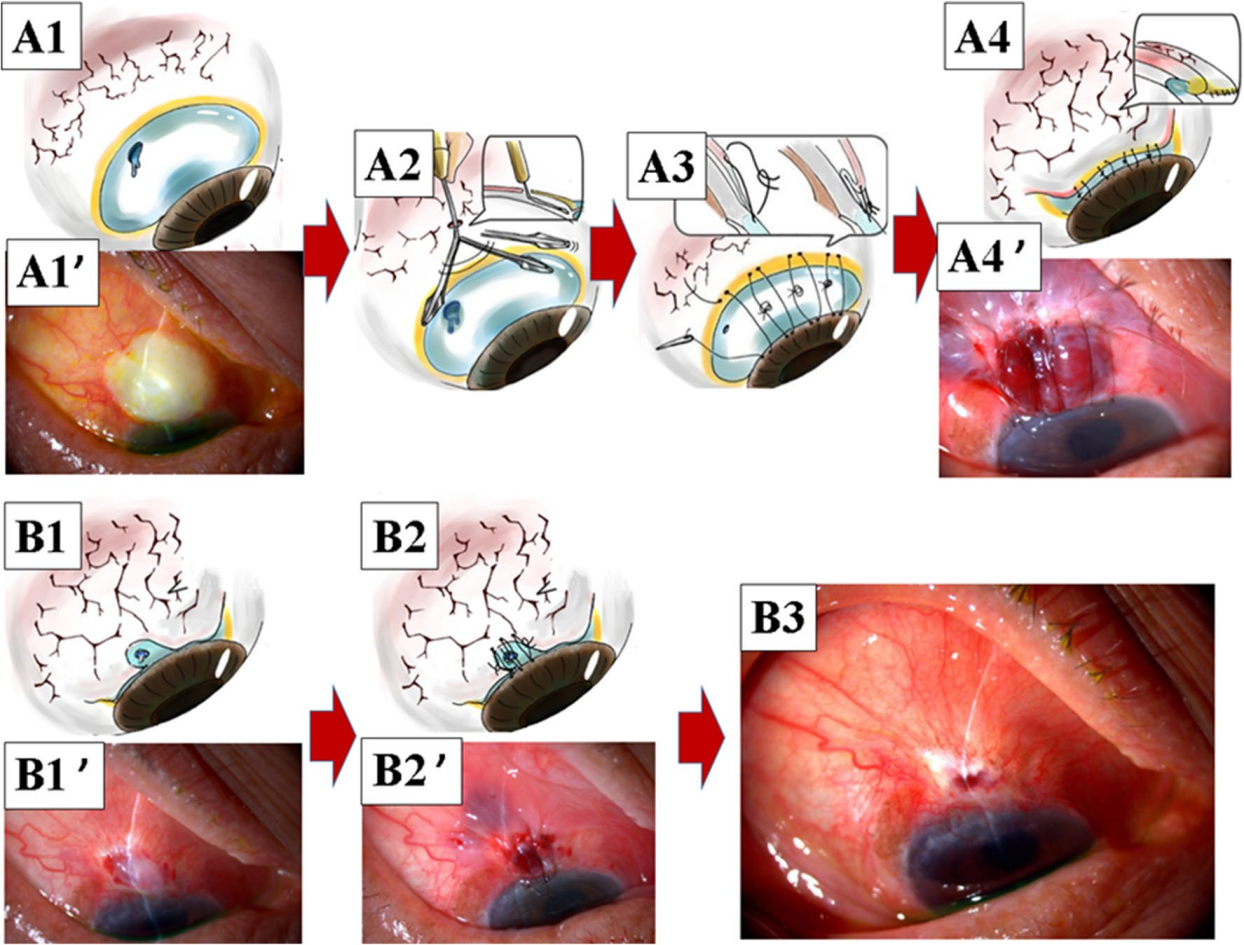
Bleb plication for the patient shown in Fig. 4. (**A1,A1′**) Bleb leakage. (**A2**) Needle bleb redirection to float the conjunctiva away from the sclera. (**A3**) Multiple O-shaped sutures. (**A4,A4′**) Completion of bleb plication. (**B1,B1′**) After the first bleb plication, the ischemic conjunctiva was decreased, but still present, and bleb leakage may still remain. (**B2,B2′**) Bleb plication was repeated. (**B3**) If the leak remains, bleb plication can be repeated, as shown in **B1**. Ultimately, the ischemic conjunctiva is covered with normal conjunctiva.

As already detailed in our previous report^27^, the bleb plication method was performed in two-steps:

1. The conjunctiva was floated away from the sclera as extensively as possible around the ischemic conjunctiva (the ischemic conjunctiva was already floated) toward the fornix, using a bleb knife (Bleb Knife II Bent 1.0; Kai Industries, Japan) (Fig. 1A1, A1′, and A2). No solution was injected.
2. Multiple (4–6, according to the size of the ischemic area of the conjunctiva) “O-shaped” sutures were applied between the non-ischemic conjunctiva just at the fornix to the ischemic bleb and corneal limbus, using 10–0 nylon (MANI suture nylon; MANI, Japan). The ischemic conjunctiva was not removed, but undermined beneath the advanced non-ischemic conjunctiva (Fig. 1A3, A4, and A4′).

The patients were given 1.5% levofloxacin ophthalmic solution (4 times/day), 2% rebamipide ophthalmic solution (2 times/day), and 1% brinzolamide solution (2 times/day for between 2 and 4 weeks).

In some cases, the operated non-ischemic conjunctiva may not reach the corneal limbus despite the use of very tight 10–0 nylon sutures, because of the limited mobility of the conjunctiva (and Tenon’s tissue) at the fornix side of the ischemic conjunctiva (Fig. 1A4′). However, even in such a case, leakage is not observed because the tight 10–0 nylon sutures change the flow of the aqueous humor under the conjunctiva toward the fornix. The 10–0 nylon sutures usually become loose within 1 week. In that case, when leakage is still observed (Fig. 1B1 and B1′), the bleb plication method can be repeated as many times as needed until the leakage stops permanently (Fig. 1B2, B2′ and B3). Leakage may no longer be observed even without complete coverage with the non-ischemic conjunctiva, because the ischemic bleb is already covered by the conjunctival epithelium. At least, the ischemic bleb is reduced in size each time, and hence, the non-ischemic conjunctiva can finally reach the corneal limbus.

**Figure 2:**
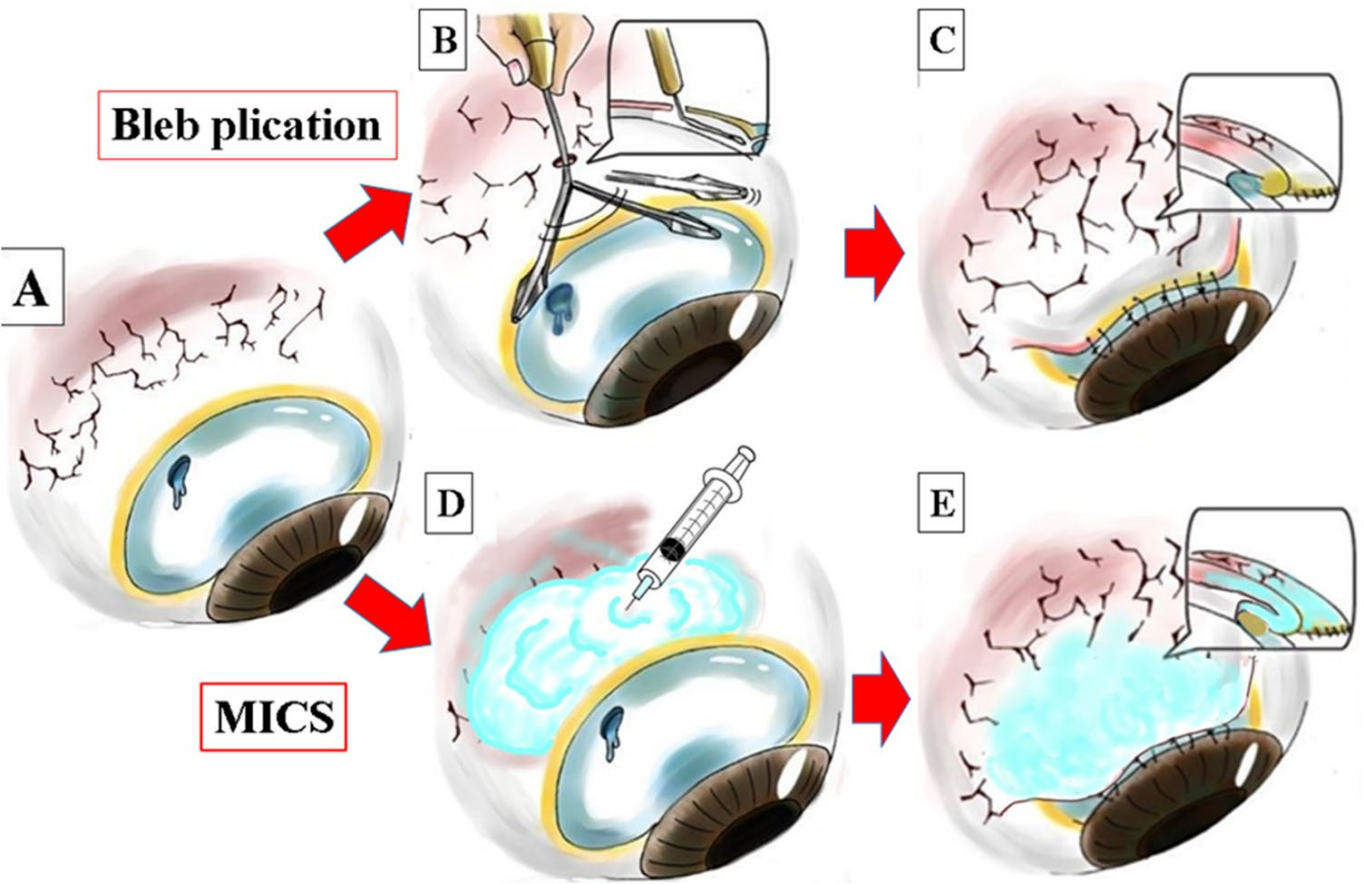
The difference between bleb plication and MICS. (**A**) Bleb leakage from a large ischemic bleb. (**B**) Bleb needle redirection to float the conjunctiva away from the sclera. (**C**) The conjunctiva is now mobile and can be plicated (sutured to the corneal limbus). As a result, the bleb is covered by the non-ischemic conjunctiva. (**D**) Subconjunctival anesthetic is injected behind the bleb. (**E**) The bleb is plicated; however, it can still be covered by the ischemic conjunctiva. MICS: incision-free minimally invasive conjunctival surgery.

A comparison of bleb plication and MICS is shown in Fig. 2A–E. In short, in bleb plication, bleb needle redirection is performed, and as a result, the bleb can be covered by the non-ischemic conjunctiva. This also enables its application to a case with tight adhesion of the conjunctiva to the sclera around the bleb.

The clinical details collected in this study were age, sex, type of glaucoma, antifibrotics used, primary surgery, time from surgery to the onset of bleb leakage, previous attempts to treat bleb leakage, the number of bleb plications performed, best corrected visual acuity (VA) and astigmatism before and after treatment using the cross- cylinder technique, intraocular pressure (IOP) before and after treatment using the Goldmann tonometry, and the medication score before and after treatment. Bleb plication was repeated until the leakage stopped. All eyes were followed up for at least 6 months after the final bleb plication surgery.

## Results

The basic demographics of the participants are summarized in Table 1. The study group consisted of 7 men and 4 women, and the mean age at treatment was 67.0 ± 15.9 (mean ± standard deviation [range: 43–94]) years. The diagnoses of the patients were: primary open-angle glaucoma (n = 7), normal tension glaucoma (n = 3), and primary closed-angle glaucoma (n = 1). All patients had trabeculectomy with either MMC (9 eyes) or 5-FU (2 eyes) as an antifibrotic agent. The mean onset of bleb leakage from the last trabeculectomy was 161.9 ± 53.0 (range: 120–244) months. All patients were Japanese. Patient 7 had a recurrent bleb leakage after conjunctival advancement^18^, and severe conjunctival scarring and impractical conjunctival mobilization were observed.

Table 2 shows the other ocular conditions and outcomes of the patients. Mean pre-operative IOP was 3.2 ± 4.1 mmHg (medication score: 0.5 ± 0.9), and the mean final IOP was 11.9 ± 2.8 mmHg (medication score: 1.5 ± 1.3). Bleb plication was needed 2.2 ± 1.5 times on average to achieve final sealing of the leakage. No patient had considerable deterioration of VA. Elevated IOP was observed in 1 eye (Patient 5) and bleb needle redirection was needed. The follow-up period (from the last bleb plication to final observation) was 16.7 ± 8.0 (range: 6–30) months after final bleb plication. The medication score was counted as 1 for each of the eye drops of prostaglandin analogues, β-blocker, carbonate dehydratase inhibitor, α2 receptor agonist, and ROC kinase inhibitor. There was no patient used the oral acetazolamide. The recurrence of bleb leakage was not observed in any eyes in the follow-up period. There was no significant difference in the astigmatism power, between before and after the surgery (Paired Wilcoxon test, p = 0.69).

**Table 2:** Ocular conditions and outcomes.

Patient No	Times of plication	Length of follow up after bleb plication (months)	Before plication				After plication			
			VA	Astigmatism power	IOP	Medication score	Final VA	Astigmatism power	Final IOP	Medication score
1	1	6	20/1,000	3	2	0	20/100	3	15	2
2	2	13	20/75	2	2	0	20/75	2	12	0
3	2	12	20/20	1.75	4	2	20/20	1.75	15	2
4	3	25	20/800	0	12	2	20/800	0	7	2
5	2	28	20/40	2.5	0	0	20/75	2	15	4
6	1	30	20/16	2	10	0	20/16	1.5	11	2
7	2	13	20/40	0.5	0	0	20/25	1.25	12	0
8	4	12	20/75	2	2	0	20/40	1.25	10	0
9	3	18	20/50	3	1	0	20/100	3.5	14	2
10	2	19	20/200	3.5	3	2	20/200	4.5	8	2
11	2	8	20/100	4.5	0	0	20/200	4.5	12	0

VA: visual acuity, IOP: intraocular pressure in mmHg.

**Figure 3:**
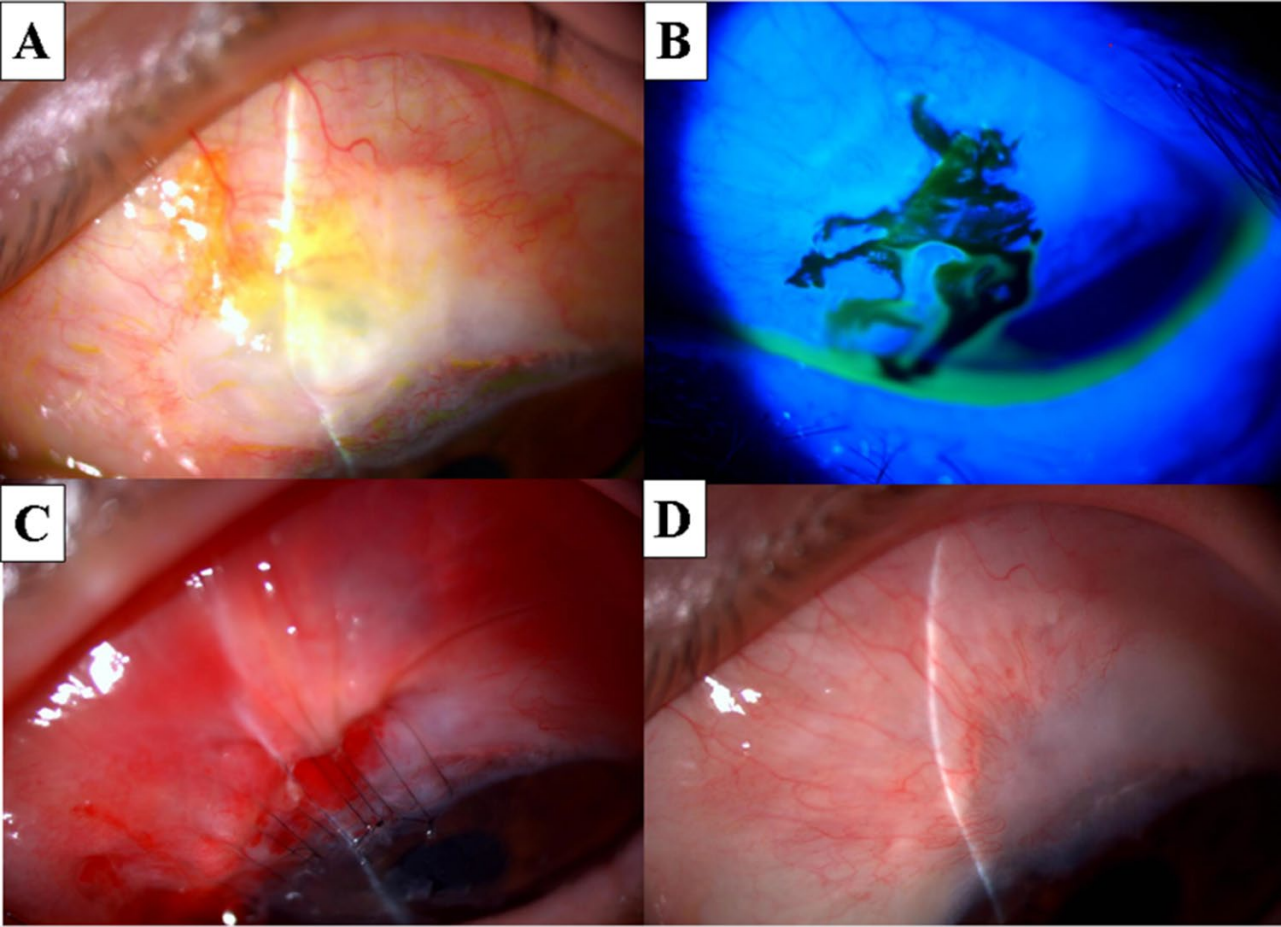
A case with normal tension glaucoma (43-year-old female, left eye: Patient 6 in Tables 1 and 2). Trabeculectomy with MMC was performed 10 years earlier. (**A**) Bleb leakage from a large ischemic bleb. (**B**) A positive Seidel test. (**C**) After initial bleb plication. (**D**) We performed the bleb plication method twice. The leakage was sealed and the ischemic conjunctiva was much decreased (at 3 months after bleb plication). MMC: Mitomycin C.

Figure 3 shows a 43-year-old female patient with normal tension glaucoma in the left eye (Patient 6 in Tables 1 and 2). Trabeculectomy with MMC was performed 10 years earlier. Slit-lamp examination revealed a positive Seidel test resulting from an ischemic bleb. IOP was 7 mmHg. Following unsuccessful bleb needle redirection and medical treatment, the bleb plication method was performed twice with an interval of a week. Consequently, the leakage was sealed (followed up for 18 months). No ischemic changes were observed in the advanced non-ischemic conjunctiva.

**Figure 4:**
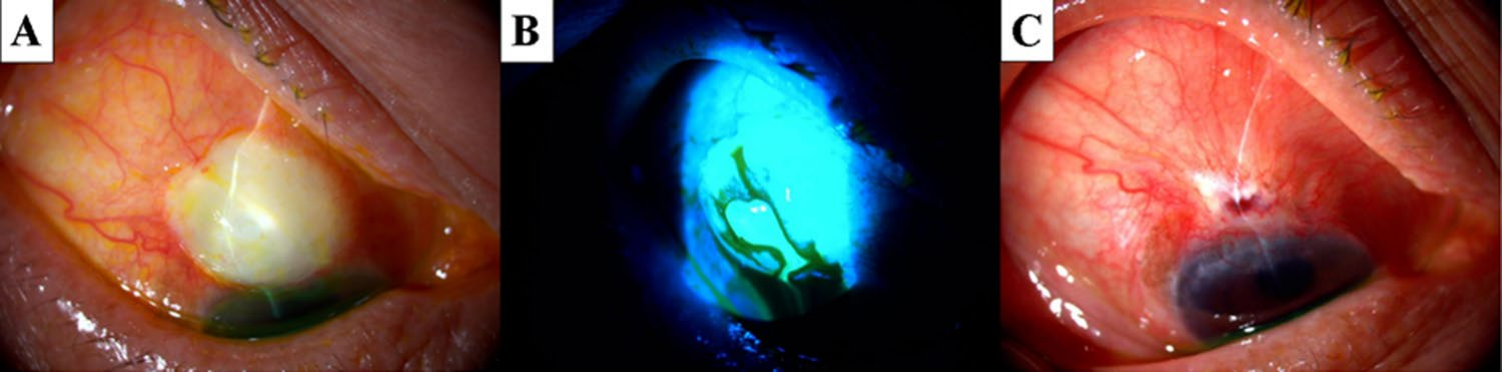
A case with primary open-angle glaucoma (83-year-old male, right eye: Patient 5 in Tables 1 and 2). Trabeculectomy with MMC was performed 14 years earlier. (**A**) An ischemic bleb surrounded by tight adherent conjunctiva. (**B**) Fluorescein staining revealed a positive Seidel test in the ischemic bleb. (**C**) The leak was sealed and the ischemic conjunctiva was reduced (at 3 months after bleb plication). MMC: Mitomycin C.

Figure 4 shows an 83-year-old male patient with primary open-angle glaucoma in the right eye (Patient 5 in Tables 1 and 2). Trabeculectomy with MMC was performed 14 years earlier. Slit-lamp examination revealed a positive Seidel test resulting from an ischemic bleb. IOP was 2 mmHg. Following unsuccessful bleb needle redirection and medical treatment, the bleb plication method was performed twice with an interval of a week. Consequently, the leakage was sealed (followed up for 24 months). No ischemic changes were observed in the advanced non-ischemic conjunctiva.

**Figure 5:**
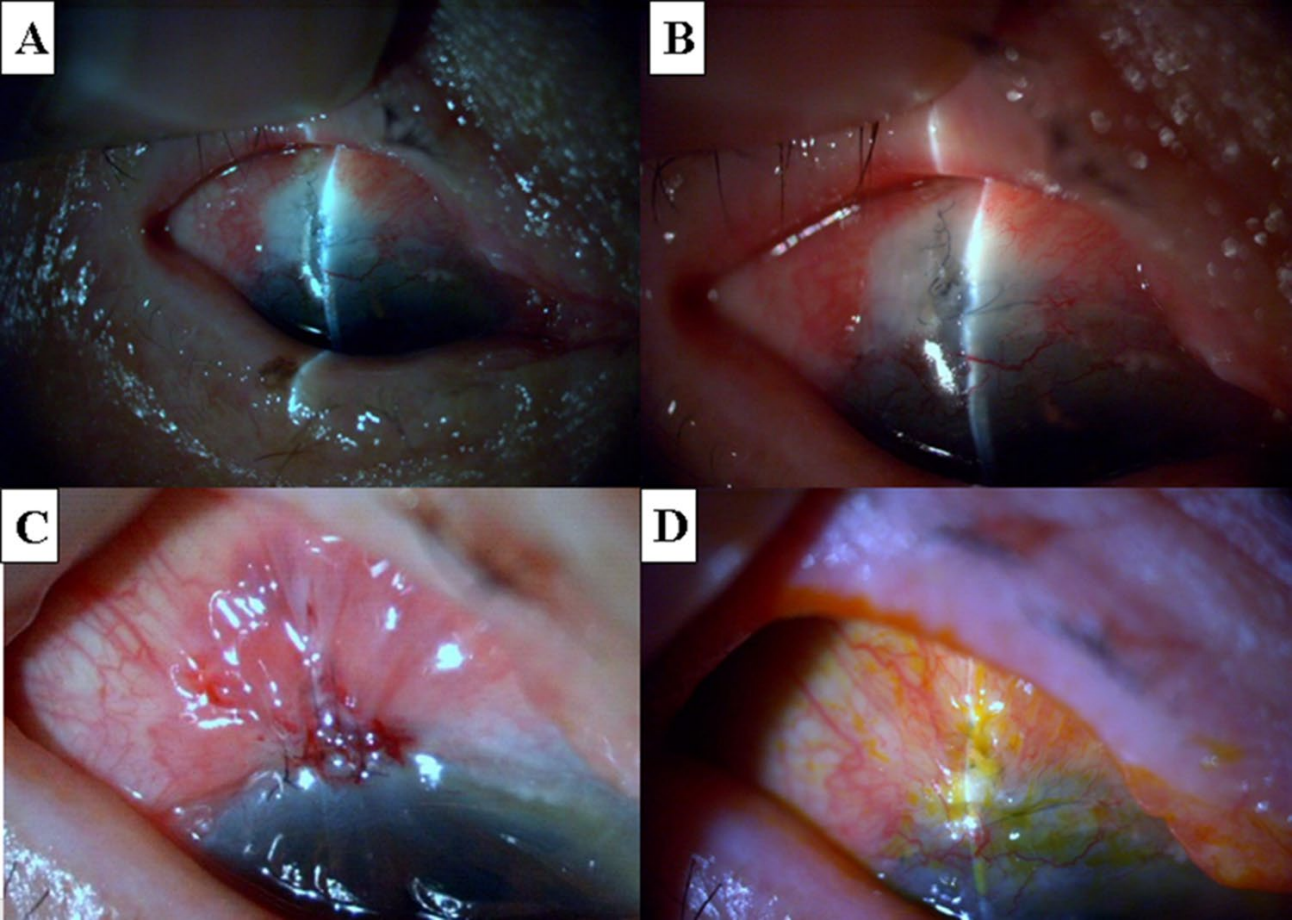
A case with primary open-angle glaucoma (94-year-old male, right eye: Patient 7 in Tables 1 and 2). Trabeculectomy with MMC was performed 14 years earlier, and conjunctival advancement was administered 4 years earlier. (**A**) Bleb leakage was sealed by conjunctival advancement 4 years earlier. (**B**) Recurrence of bleb leakage from the ischemic bleb. The mobility of the conjunctiva was poor and ischemic conjunctiva still remains in the bleb. (**C**) After bleb plication. (**D**) The leakage was sealed and the ischemic conjunctiva was reduced (at 4 months after bleb plication). MMC: Mitomycin C.

Figure 5 shows a 94-year-old male patient with primary open-angle glaucoma in the right eye (Patient 7 in Tables 1 and 2). Trabeculectomy with MMC was performed 14 years earlier. As treatment for bleb leakage, conjunctival advancement was performed 4 years earlier. However, the leakage recurred and bleb plication was performed twice with an interval of a week. The ischemic conjunctiva remained in the bleb and the mobility of the conjunctiva was poor.

## Discussion

In the present study, we report the outcome of the bleb plication method with a relatively long follow-up period (at least 6 months) in 11 consecutive eyes with bleb leakage, including those with severe conjunctival scarring and impractical conjunctival mobilization. In bleb plication, the ischemic conjunctiva is not removed, but is undermined beneath the advanced non-ischemic conjunctiva. Bleb leakage was successfully sealed in all cases without any serious complications. During the follow-up period, bleb leakage did not recur because the non-ischemic conjunctiva was used to cover the ischemic bleb.

Bleb leakage is a serious complication of glaucoma filtering surgery with MMC or 5-FU, and often develops into an ischemic bleb^6^. Many medical and surgical methods have been proposed to seal the leakage from a bleb, such as aqueous suppressants, e.g., a β-adrenergic blocking agent in combination with a carbonic anhydrase inhibitor^14^. A large diameter bandage contact lens is another option; however, this approach depends on the location of the leakage^14,15^. A glaucoma shell works by applying high resistance to excessive aqueous humor run-off through a leaking wound, and promotes conjunctival epithelial closure by approximating the wound edges^17^. In addition, cyanoacrylate tissue glue^21^ and a collagen shield^16^ may be useful to seal the leakage. Bleb needle redirection^19^ is used to increase the size of a bleb, and thereby reduce the pressure on the ischemic conjunctiva. Bleeding within a bleb also helps to seal the leakage, similarly to subconjunctival blood injection^29^. However, one of the fundamental problems associated with bleb needle redirection is that the ischemic conjunctiva remains, even after this treatment, which leads to the recurrence of bleb leakage. There are other options when the conjunctiva is insufficient or ischemic, such as autologous conjunctival patch grafting^22^, amniotic membrane transplantation^23^, scleral patch graft^24^, corneal patch graft^25^, and fascia autograft^26^. Despite these treatments, bleb leakage often cannot be treated successfully and treatment should be upgraded to a more invasive approach, such as conjunctival advancement^18^ and MICS^27^.

Both conjunctival advancement^18^ and MICS^28^ attempt to cover an ischemic bleb with the non-ischemic conjunctiva. However, surgeons are often faced with a shortage of conjunctiva after the removal of the ischemic conjunctiva. In MICS, the ischemic conjunctiva is not removed; however, this method cannot be applied to a case with severe conjunctival scarring or in whom conjunctiva mobilization is impractical^27^. A merit of the currently proposed method of bleb plication is that the conjunctiva can be floated by bleb needle redirection, even if the mobility of the conjunctiva is poor, and as a result, the mobile conjunctiva can be used to cover the ischemic conjunctiva. In the current study, this method was free of serious complications, because the non-ischemic conjunctiva is simply sutured to the corneal limbus without removing the ischemic bleb. All of the steps for bleb plication can be performed without special equipment. This method would be effective even in eyes with a narrow eyelid cleft or severe conjunctival scar, as is often seen in Japanese patients. Another merit of the bleb plication approach is that it can be repeated as many times as needed, because the conjunctiva can be floated by bleb needle redirection prior to the plication of the bleb. In addition, the conjunctiva is not excised, and hence there is no concern that the conjunctiva will run out. Indeed, in one patient in our study, bleb leakage recurred at 4 years after conjunctival advancement; however, the leakage was sealed after performing bleb plication twice (Patient 7, Fig. 5).

There are other proposed methods to treat bleb leakage by covering the ischemic conjunctiva with the non-ischemic conjunctiva, similarly to the bleb plication method. The method reported by Rai et al.^30^ proposed suturing the mobile conjunctiva (at the fornix side) to the limbus. This method for bleb leakage is performed just after trabeculectomy, and hence it is assumed that the conjunctiva is mobile. Conversely, our method can be applied to leaking ischemic blebs after considerably long periods of time. As a result, the conjunctiva at the fornix side of the bleb has already adhered to the sclera, and the bleb needle redirection technique is needed to float the conjunctiva prior to its plication. Indeed, as shown in the current study, the bleb plication method was successfully performed even in eyes with severe conjunctival scarring and impractical conjunctival mobilization (Patient 7, Fig. 5). Melo et al.^31^ also reported bleb de-epithelialization with conjunctival advancement, but a conjunctival peritomy is needed with this approach, in contrast to the bleb plication method.

In the current study, there was no eye experienced serious complications, such as infectious blebitis and endophthalmitis. In addition, due to the method of the bleb plication surgery, we were not suffered from the shortage of conjunctiva which is often experienced in the conjunctival advancement. These results suggested the safety of the currently proposed method, however a careful observation is needed in the post-operative IOP, since there were some eyes which had IOP as high as 15 mmHg. This level of IOP may not be identical after trabeculectomy in eyes with NTG. These values were much higher than those before bleb plication which is disadvantageous for the progression of glaucoma per se, however the very low IOP before the surgery (between 0 and 12 mmHg) was the result of the leakage from bleb, and such eyes are in a serious risk of infectious blebitis and endophthalmitis. As a result, these eyes can be considered as being in a relatively safer condition after the bleb plication compared to before the surgery, despite the relatively elevated IOP. Although there was no difference in the VA between before and after the surgery, there were 3 eyes showed the deterioration of VA. In 2 of these cases, the astigmatism power was not different before and after the surgery, however there is a possibility that irregular astigmatism was increased in these cases. A further investigation shedding light on this issue should be conducted in a future study.

As a limitation of the current study, there was not a comparison group, such as MICS-treated group. A future study would be needed to compare the safety and effectiveness of the currently proposed bleb plication method and such other methods.

In this study, we reported the consecutive outcomes of the bleb plication method to seal a leakage from an ischemic bleb. With a follow-up period of at least 6 months, this method was found to be safe and effective in all cases.
